# Substance Abuse Counselors’ Recovery Status and Self-Schemas: Preliminary Implications for Empirically Supported Treatment Implementation

**DOI:** 10.4303/jdar/235982

**Published:** 2016-08-12

**Authors:** Elizabeth M. Nielson

**Affiliations:** Rory Meyers College of Nursing, New York University, 433 1st Avenue, New York, NY 10010, USA

**Keywords:** substance abuse counselors, recovery status, empirically-supported treatment, self-schema, addiction

## Abstract

**Purpose:**

The purpose of this paper is to better understand the relationship between substance abuse counselors’ personal recovery status, self-schemas, and willingness to use empirically supported treatments for substance use disorders.

**Methods:**

A phenomenological qualitative study enrolled 12 practicing substance abuse counselors.

**Results:**

Within this sample, recovering counselors tended to see those who suffer from addiction as qualitatively different from those who do not and hence themselves as similar to their patients, while nonrecovering counselors tended to see patients as experiencing a specific variety of the same basic human struggles everyone experiences, and hence also felt able to relate to their patients’ struggles.

**Discussion:**

Since empirically supported treatments may fit more or less neatly within one or the other of these viewpoints, this finding suggests that counselors’ recovery status and corresponding self-schemas may be related to counselor willingness to learn and practice specific treatments.

## 1. Introduction

There exists a troublesome gap between addiction science and the substance use disorder (SUD) treatment that is available to the public [1,2,3]. The substance abuse treatment field has been criticized for failing to offer appealing treatment options [1] and for continuing to use unproven or even discredited practices [4]. Indeed, treatment methods with documented iatrogenic effects are commonplace in the addictions field [4,5,6]. Despite the fact that empirically supported treatments (ESTs) for SUDs exist, the majority of individuals treated for SUDs in the US receive services that are not empirically supported or have less empirical support than other options [7]. For some individuals, continuing to misuse substances is preferable to enduring SUD treatment because goals are inflexible, such as mandatory abstinence goals [8], or treatment involves coercion or confrontation [9]. Efforts to improve adoption of ESTs have looked at organizational barriers [10], program staff attitudes [11], and the professional credentialing process [12].

Substance abuse counselors are a diverse group with a range of education levels and backgrounds. In many states it is possible to become a counselor with as little as a general high-school equivalency diploma and some counseling-specific coursework [12]. In any educational or credentialing category, a clinician may or may not self-identify as personally being in recovery from addiction. Counselor recovery status is the single most researched factor in the diverse population of substance abuse treatment providers [13]. Historically, substance abuse treatment centers were founded and run by individuals who were in recovery from addiction themselves, and largely promoted the idea that recovering addicts were the best—if not only—ones to provide psychosocial support to people with SUDs [14]. Although the field of substance abuse counseling has evolved to include professionals without personal histories of addiction and recovery, and research shows that nonrecovering counselors are as effective as their recovering counterparts [13], misconceptions surrounding the relative competence and preparedness of recovering and nonrecovering counselors may still be found among counselors, patients, and the public.

Several findings point to differences in the ways that recovering and nonrecovering counselors may understand addiction, conceptualize their patients’ problems, and ultimately perform their jobs. For instance, Lawson et al. found that recovering counselors were more likely than nonrecovering counselors to diagnose alcoholism when given the same information about a potential patient [15]. Stöffelmayr et al. found that recovering counselors used a wider range of treatment techniques and goals than their nonrecovering colleagues [16]. Curtis and Eby found that recovering counselors had higher affective professional commitment to their work than nonrecovering counselors; however recovery status did not predict organizational commitment [17]. Olmstead et al. suggested that the comparatively lower salaries earned by recovering counselors could be due to their sense of personal commitment to the field, which results in willingness to take lower-paying positions even when they have a relevant college or graduate degree [18]. These existing findings on differences by recovery status are of increasing importance as researchers recognize the role of diverse counselor factors in the dissemination of ESTs [19].

The reasons for the continued use of unproven SUD treatments are unclear and probably include several factors such as organizational structure and funding sources [20]. Research shows that some treatment providers have theoretical misgivings about implementing ESTs that conflict with their more traditional beliefs about the nature of SUDs [20,21,22,23]. For instance, counselors have been found to discount the effectiveness of contingency management because of a belief that it will “send the wrong message,” to clients [20].

## 2. Methods

The present exploratory qualitative interview study was conducted to gather preliminary data on the relationship between substance abuse counselors’ beliefs about the nature of addiction and willingness to learn and implement ESTs. The study was designed to explore substance abuse counselors’ perceptions, attitudes, and lived experiences, and to answer the following five specific research questions:

1. how do substance abuse counselor beliefs about the etiology and treatment of SUDs evolve to enhance the acceptance of ESTs within the broader context of evidence-based practice?
2. what type of evidence for a given treatment method will counselors with more (or less) traditional (12-step) beliefs find acceptable?
3. how do counselors see the role of personal experience of addiction/recovery (or lack of) or of being a concerned significant other (CSO; having been affected by a close friend or family member who struggled with an addictive disorder [24]) in forming beliefs about addiction?
4. how does change in beliefs about the etiology of addiction result in increased willingness to engage in evidence-based practice?
5. what can be done to promote counselor belief change toward contemporary conceptualizations of addiction?

The study was approved by the Institutional Review Board (IRB) at Walden University and issued a Certificate of Confidentiality by the National Institute on Alcohol Abuse and Alcoholism (NIAAA).

### 2.1. Recruitment

Participants were recruited through the researcher’s network of professional contacts, including professional email lists and word of mouth. In order to meet inclusion criterion, participants had to be clinicians who primarily treated SUD in their practice. The study did not limit participation to any specific credentialing category or level, education level, or practice setting.

### 2.2. Instruments

A measure of counselors’ agreement with the 12-step/AA philosophy was taken using the 12-step beliefs scale [25]. This scale been used consistently in research on substance abuse counselor beliefs making it somewhat of a standard way to assess this dimension in counselors [21,26,27,28, 29,30]. Scores were divided into categories of low, medium, and high 12-step beliefs for analysis.

Interviews were conducted using a semistructured interview guide [31]. The guide allowed for flexibility while ensuring that the same topics would be covered with each participant. Two independent reviewers provided feedback on the interview guide prior to its use in the study. All interviews were audio-recorded and transcribed by the researcher. Transcripts were imported into NVivo qualitative data analysis software and then coded for themes based on the study’s research questions and emergent trends noted while reading and coding interviews [32].

### 2.3. Data analysis

Interview data were analyzed following a phenomenological approach [33,34]. First, transcripts were classified in broad categories indicated by the existing literature on substance abuse counselors’ beliefs, clinical orientations, and experiences of personal recovery [35]. Counselors’ comments were then coded as they referred to and provided understanding of the phenomenon of interest such as personal recovery, EST implementation, forming 12-step beliefs, navigating institution or policy issues, and being a CSO [13,25,36,37]. Initially, 10 transcripts were coded to create preliminary findings, with two transcripts held back for comparison and to ensure saturation. When preliminary findings were reached, these were summarized and sent to all participants who elected to provide feedback and could be reached at their preferred contact email or phone number (*n* = 10). Feedback was received from 6 (50%) participants and incorporated into the final analysis.

## 3. Results

The purpose of phenomenological research is to create a situation in which findings come from the interviewee’s actual lived experience, with no predetermined answers provided by the researcher. For this reason, qualitative research is often used to discover trends and generate hypotheses that can be further refined and elaborated in future quantitative studies. The findings reported here emerged during analysis of transcribed interviews and were not responses to any specific question posed by the study.

### 3.1. Participants

Twelve participants enrolled in the study. Ten participants completed interviews by telephone, and two chose to be interviewed in person. Interviews were scheduled according to participants’ choice of time and location. The two participants who completed interviews in person requested that the interviews be conducted in their homes for privacy and convenience. Participants who completed phone interviews did so either from their offices or home, depending on where they could find the most privacy. Five participants were located in New York State and one was from New Jersey. The remaining six represented the Pacific Northwest, Midwest, and Southern regions of the US. All phone interviews were conducted from the researcher’s private home office in New York State. Only one interview was interrupted briefly when the participant received another call and put the interviewer on hold for approximately five minutes.

Half of the participants self-identified as having a personal history of addiction (recovering counselors), and half self-identified as not having a personal history of addiction (nonrecovering counselors). Nine counselors identified as being a CSO. Participants had between 3.5 and 40 years of experience in the field (*M* = 12.46, SD = 10.35). There were four female participants and eight male participants. Ages ranged from 31 to 68 years, with a mean age of 51.75 years (SD = 14.83929). Eleven participants identified as White/Caucasian. One specified a Jewish ethnicity. Although not intended to be representative, the sample was similar to samples for other studies of counselor attitudes and beliefs in terms of recovery status and education level [19]. Table 1 describes the study sample.

### 3.2. 12-step beliefs scores

Scores on the 12-step beliefs scale were averaged to create a single numerical score between 1 and 7. These scores were then divided into three categories; low 12-step beliefs (scores 1–2.99), medium 12-step beliefs (scores 2.0–5.0), and high 12-step beliefs (scores 5.01–7.0). Low scores indicated more contemporary beliefs and high scores more traditional beliefs. Three counselors fell in the low category, five in the medium category, and four in the high category. The distribution of 12-step beliefs categories is described in Table 1.

### 3.3. Education and credentials

Six participants had a master’s degree, three had a bachelor’s degree, two had a doctorate, and one had some college but no degree. The two doctoral level participants were not in recovery, were male, identified as CSOs, and had medium and low 12-step beliefs scores. Of the six master’s level counselors, half were in personal recovery, half were female, and two-thirds identified as CSOs. Two of the master’s level counselors had high 12-step beliefs, two had medium beliefs, and two had low beliefs. Among the three BA level participants and the one participant with some college experience, there were two high and two medium 12-step belief scores; three out of the four were male, in recovery, and CSOs; and the fourth was female, not in recovery, and not a CSO. Eleven participants reported licenses and credentials such as substance abuse counselors, social workers, and psychologists. The 12th reported certification as a yoga teacher and additional training and experience in providing empirically supported yoga- and meditation-based therapies to substance abuse treatment program participants.

### 3.4. Recovering counselors’ views

Recovering counselors in the present study tended to see persons who suffer (or suffered) from addiction and persons who do not (or have not) in two distinct and mutually exclusive categories. The view that one either is or is not an addict was espoused by recovering counselors regardless the level of 12-step beliefs or theoretical orientation. One recovering counselor with medium 12-step beliefs summed up this position with the succinct statement, “Once a pickle, never a cucumber.” Another recovering counselor, also with medium 12-step beliefs, clearly stated a belief in a qualitative difference between those who have developed an addiction and those who do not. “When you cross that line that’s where it crosses into addiction, that’s where it becomes physical dependency or psychological dependence.” These statements demonstrate a belief that once a person develops an addiction they are qualitatively and permanently different from those who do not have an addiction.

By extension, recovering counselors placed themselves in the same category as their patients, and some expressed the idea that this gave them an advantage in their work. These counselors attributed a variety of benefits to their recovery status including improved empathy and compassion for patients, having better boundaries and insights, and being able to do a better job than nonrecovering counselors. Some of the counselors in recovery saw themselves as having a better attitude toward, and connection with, their patients than their nonrecovering colleagues. These counselors, for the most part, believed that there was something unique about addiction that translated into recovering counselors being better prepared to treat addiction. One counselor in describing the role of his personal recovery in building a connection with his patients stated:

The personal experience piece gives me the evangelical tilt in my approach. People really know that I know. When you hear me talk about this to a group of people it’s pretty powerful because I really know, you know it’s one thing when you’re talking about something that you’re not sure of, that makes sense to you based on an equation and something else when you talk about something that you know works because its worked for you. Big difference, I think… I can be evangelical about it because I know how it works.

Another talked about how her nonrecovering colleagues were prone to feeling frustrated with patients:

I think some of my colleagues who don’t have experience with addiction, although they are very highly skilled a lot of them, they might get a little more, oh my gosh, tired, you know? Like “oh geez there he goes again.”

And a third recovering counselor discussed how her personal history of recovery acted as a protective factor against becoming burnt out or taking patient failures personally:

I understand that I may or may not help this person right now, and I’m just going to do the best that I can and if they do something, say when my clients are in trouble I don’t take it personally, I don’t get upset.

**Figure 1:**
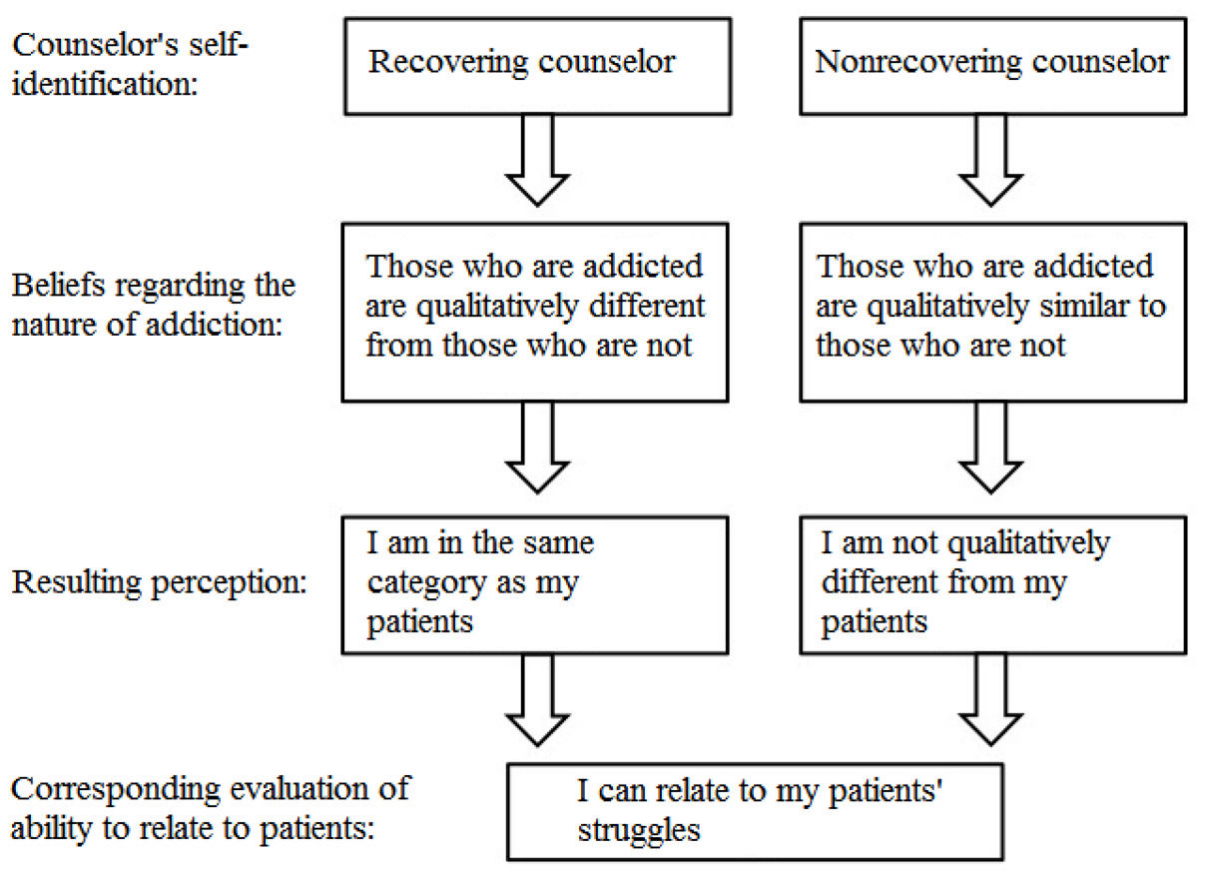
Pathways to relating to patient struggles.

The above examples demonstrate that recovering counselors attribute a special ability to connect with patients and better attitudes toward patients to their personal history of recovery. Figure 1 illustrates this as one of two pathways counselors in this study took to feel they could relate to their patients’ struggles.

### 3.5. Nonrecovering counselors’ views

Nonrecovering counselors tended to see themselves and their patients on a continuum. They described addiction as one of several varieties of suffering to which they could relate even though their own experiences of suffering had different causes. Several of the nonrecovering counselors acknowledged having had the *potential* to develop an addictive disorder themselves, but felt that because of their own determination, choices, or other factors in life they had never fully developed a problem. These counselors seemed to see suffering as a universal human condition with less emphasis on the cause and more on the experience.

For instance, one nonrecovering counselor discussed her experience of struggling to get out of a difficult situation in young adulthood in order not to develop a problem with drugs or alcohol. She acknowledged the potential or developing such problems had she not changed her situation early in life:

Had I stayed in the circumstances that I was in, I mean I graduated early, I left the area, because I *knew* it was a bad scene, I was around bad people, and had I not, I don’t know, I may have been weak enough to hang in there and who knows what would have happened, but I had at that point in my life a greater desire to do something with myself. I wanted to learn.

A major theme among the comments of nonrecovering counselors’ comments on beliefs about addiction was that there is no qualitative difference between those who are in recovery and those who are not. Nonrecovering counselors thought of themselves as more similar to their patients than dissimilar with regards to overall experience of suffering and struggle. For instance, a nonrecovering counselor noted that not having a history of addiction recovery did not equate not having had any difficulties or need for personal work as some might think. Referring to a patient, he stated, “Everything he’s done in his 12-step I’ve done in my own therapy somewhere. So in that sense I’m not really asking them to do anything I haven’t done.” In this study sample none of the nonrecovering counselors expressed concern that they would be unable to do as good a job as their recovering colleagues, nor did they seem to see themselves as categorically different from their patients.

In sum, both recovering and nonrecovering counselors saw themselves as similar to, and being able to relate to, their patients. Recovering counselors recognized those with addictive disorders as categorically different from those without them, and placed themselves in the same category as their patients. Nonrecovering counselors, for the most part, did not draw categorical lines between those with addictive disorders (patients) and those without (themselves). These two distinct pathways seemed to lead to similar ends; feelings of understanding and empathy for patients’ struggles.

## 4. Discussion

### 4.1. Self-schemas and the DSM-5

The DSM-5 represented a major shift in the diagnostic system for addictive disorders [38]. The interviews for this study (conducted in June and July of 2013) came just one month before the publication of the DSM-5 and make for timely documentation of the attitudes exposed by counselors as this transition went into effect. In the previous edition of the DSM, SUDs were divided into two categories, substance abuse and substance dependence [39]. Dependence was described as a more severe form of addiction, involving physiological tolerance and withdrawal where appropriate. The system was designed to classify patients categorically as either having or not having an additive disorder.

With the DSM-5 the entire diagnostic system for addictive disorders changed to a continuum model, making the changes to the diagnostic system for addictions arguably the most significant changes to any one diagnostic category. With regards to the substance abuse counselors’ views described in the present study, the DSM-5’s continuum model is more closely aligned with the views of the nonrecovering counselors, or those who did not identify as ever having developed an *addicted* self-schema [40]. The addicted self-schema includes extremely negative self-attributions and negative affective states, and recovery from addiction involves the replacement of the addicted self-schema with a more positive *nonaddicted* self-schema [40]. Furthermore, the continuum model supports the notion that those who meet criterion for a mild SUD might never meet the criterion for a severe disorder, and it is possible that one could meet any level of diagnostic criterion with or without having developed an addicted self-schema. Therefore, the presence of an addicted self-schema cannot be seen as diagnostic in and of itself, nor can it neatly determine which individuals are in need of substance use treatment.

### 4.2. Implications for training in ESTs

One of the main issues the addiction treatment field has to face is the gap between research and practice. While cutting-edge, science-based, ESTs are available, the transfer of these interventions to community-based settings is notoriously slow. The result is that many patients receive suboptimal care with treatment methods that are outdated, ineffective, or even iatrogenic. Moving ESTs from the world of researchers to the world of front line counselors has been one of the fields’ top priorities in recent years yet much remains to be done.

Substance abuse treatment providers have a variety of views about what constitutes adequate and necessary experience to become a counselor and how personal experience of addiction and recovery relates to being an effective counselor. In order to provide training in ESTs to front-line counselors, training programs would do well to take these factors into account, lest the interventions should be outright rejected for failure to match the audiences’ beliefs. To present counselors with new treatment interventions backed by a wealth of research without mentioning the role of the counselor’s personal experience may imply that personal experience is not necessary or important in providing effective services and thereby alienate those who do hold this view. Likewise, treatments which implicitly (or explicitly) promote the idea of those with SUDs and those without being qualitatively different from each other or being at two points along a continuum run the risk of being rejected by counselors who believe otherwise.

### 4.3. Implications for evidence-based practice implementation

Evidence-based practice (EBP) requires the clinician to integrate knowledge of the best ESTs, the individual patients’ characteristics and preferences, and their own clinical expertise to arrive at a treatment plan that is individualized for each patient [41]. If clinicians, recovering or not, reject ESTs outright because these treatments conflict with deeply held beliefs about the nature of addiction or the role of personal recovery in qualifying someone to be a counselor they cannot be integrated into an EST model. It is up to the research community to vet, refine, and publish the best possible treatments. It is also imperative that these treatments be make these acceptable to the counselor population so that they can be implemented.

While training programs may not address such firmly held beliefs, efforts to educate counselors on the use of ESTs would do well to account for them, perhaps by screening for such beliefs and tailoring teaching to them. Additionally, some ESTs are based on theories that do not conceptualize a qualitative difference between those who have a SUD and those who do not (i.e., Mindfulness-Based Relapse Prevention) [42]. Other ESTs, such as pharmacotherapies, are based on the idea that those who suffer from addiction are qualitatively different from those who do not. A training program that assumed one or the other stance among its audience could easily be rejected by those who conceptualize the nature of addiction differently, whereas aligning training with counselor beliefs could produce increased engagement and learning. Differences between counselors’ conceptualization of addiction and that of a particular EST need not prevent those counselors from being willing to incorporate that treatment into their EBP. It is notable that in this study, regardless of whether counselors believed those with addictive disorders were categorically different from those without or not, all counselors felt they were adequately able to relate to their patients’ experiences.

## 5. Study limitations

The present study has several limitations. First, as a small, exploratory, qualitative study, the findings are not necessarily generalizable to the larger population of substance abuse counselors. They can instead be considered preliminary ideas for further exploration on a larger scale. The study is limited by its sampling method and lack of compensation, both of which favored interested counselors with adequate free time to self-select for enrollment. Furthermore, because the majority of participants were contacted through professional email lists and professional networks, the study participants may have had more interest in professional issues and evidence-based practice than average counselors.

The study of counselors based on recovery status is somewhat problematic. Addiction has been described as resulting in the activation of a cognitive self-schema, in which the individual self-identifies as an addict and processes incoming information accordingly [40]. When one self-identifies as in recovery from an addiction, the implication is that they have recognized the addict-schema and shifted to a recovery-oriented schema. This process is highly personal, variable, and eludes direct assessment. Self-identification as “in recovery” may mean different things to different people at different times, confounding its utility as a parameter in research. Furthermore, when posed as a dichotomous question, recovery status necessarily eliminates from study the rich diversity of experiences with substances that could likely be found within both groups. It is also possible to imagine many potential factors besides a counselor’s history of alcohol or drug use that could influence identification as “in recovery.” For instance, counselors may also be more or less likely to identify as “in recovery” if they attended a drug treatment program that promoted the use of the term (and adoption of the corresponding self-schema), as opposed to, for instance, recovering without formal intervention or 12-step involvement. Although counselors’ recovery status is commonly measured as a dichotomous variable, it therefore may not have the reliability of factors with consistent, objective criterion such as diagnoses or biomarkers.

## 6. Future research

Dissemination of ESTs to community-based substance abuse treatment settings remains slow and incomplete. Counselor beliefs about the nature of addiction are clearly a barrier to dissemination; one that requires further research and targeted interventions to overcome. The present study further illuminates the complexity of substance abuse counselor beliefs, both with regards to the impact of within-group recovery status differences on competence and to patient/problem conceptualization. Further research should explore the prevalence and correlates of these beliefs such that they can be anticipated within subsections of the counselor population. Such refinement may yield findings that can be used to tailor EST trainings and result in improved EPB adoption.

## Figures and Tables

**Table 1:** Participant data.

	Recovering	Nonrecovering
Male	4	4
Female	2	2
Education		
PhD		2
MA	3	3
Some College/BA	3	1
CSO	6	3
12-step beliefs		
High	2	2
Medium	3	2
Low	1	2
Total	6	6
